# Comparison of Reachable Workspace Capacity to Real-World Arm Use Performance: A Wearable Accelerometry Study on Chronic Stroke

**DOI:** 10.3390/s26175651

**Published:** 2026-09-05

**Authors:** Guillem Cornella-Barba, Natanya Gunn, Vicky Chan, David J. Reinkensmeyer, Jay J. Han

**Affiliations:** 1 Department of Mechanical and Aerospace Engineering, University of California Irvine, Irvine, CA 92617, USA; 2 Department of Physical Medicine and Rehabilitation, University of California, Orange, CA 92868, USA; 3 School of Medicine, University of California Irvine, Irvine, CA 92697, USA; 4 Department of Rehabilitation Services, Irvine Medical Center, University of California, Orange, CA 92868, USA; 5 Department of Anatomy and Neurobiology, University of California Irvine, Irvine, CA 92697, USA

**Keywords:** stroke rehabilitation, upper extremity, reachable workspace, relative surface area, wearable sensors, accelerometry, smartwatch, ADEM, real-world arm use, movement diversity, motor activity

## Abstract

**Highlights:**

**What are the main findings?**

- Reduced Reachable Workspace (RWS), quantified using the camera-derived Relative Surface Area (RSA) score, was associated with decreased arm use in daily life (ρ = 0.66), as measured with an accelerometer-based, smartwatch-derived metric in 14 individuals with chronic stroke.
- RSA was also significantly associated with the Box and Block Test (BBT), Upper-Extremity Fugl-Meyer Assessment (UEFM), and Motor Activity Log (MAL), with correlation strengths ranging from ρ = 0.57 to 0.76.

**What are the implications of the main findings?**

- Combining camera-based RWS assessment with smartwatch monitoring may help relate upper-extremity movement capacity measured in the clinic to arm movement behavior during daily life.
- RSA may provide an objective and spatially detailed measure of post-stroke arm impairment that can be interpreted alongside clinical motor assessments and patient-reported arm use.

**Abstract:**

Reachable workspace (RWS) quantifies the three-dimensional space accessible to the upper limb, but its relationship with real-world arm use after stroke remains unclear. This study examined associations between camera-based RWS and smartwatch-derived movement behavior in 14 individuals with chronic stroke. Relative surface area (RSA) was measured at baseline, after which participants wore a smartwatch on the affected arm. Smartwatch data were summarized using Gross Movement and Activity Count (GMAC), representing the proportion of wear time spent in active arm movement within a functional workspace, and Active and Diverse Exploratory Movement (ADEM), representing the proportion of wear time that was active and diverse. Clinical outcomes included the Box and Block Test (BBT), Upper-Extremity Fugl-Meyer Assessment (UEFM), and Motor Activity Log (MAL). Total RSA and several workspace regions were reduced in the paretic compared with the nonparetic limb. Greater total RSA was significantly associated with ADEM (ρ = 0.66, *p* = 0.01), while its association with GMAC was numerically weaker and non-significant (ρ = 0.47, *p* = 0.09). RSA was also significantly associated with BBT, UEFM, and MAL, with correlations ranging from ρ = 0.57 to 0.76. These findings link camera-based RWS with wrist-worn measures of real-world arm performance after stroke.

## 1. Introduction

After a stroke, persistent upper-extremity (UE) impairment remains present in nearly half of survivors [1,2]. These impairments can restrict active range of motion (ROM) and the ability to position the arm and hand throughout three-dimensional space [3], thereby limiting the performance of everyday tasks and reducing independence in daily life [1]. Impaired UE motor control can also make activities of daily living (ADLs) and self-care tasks, such as feeding, grooming, dressing, and toileting, more difficult, reduce participation in work and leisure activities [4], and ultimately affect quality of life (QoL) [5]. Despite the importance of upper-extremity recovery, many clinical assessments still rely on ordinal rating scales [6,7], isolated strength measures [8], or task-specific tests [9] that may not fully capture the complex three-dimensional nature of arm function [10] or provide enough sensitivity to detect meaningful changes over time [11].

Reachable Workspace (RWS) helps address this measurement gap by providing an objective, reliable, and spatially comprehensive measure of upper-extremity movement capacity [12] by using motion capture to estimate the portion of a shoulder-centered workspace that an individual can access with the arm, normalized to arm length [10,13]. This capacity is summarized as relative surface area (RSA) [12,14] and provides a continuous metric of arm mobility which has been shown to correlate with clinical impairment, functional ability, clinician-reported outcomes, and patient-reported outcomes across multiple upper-extremity disorders [12]. RWS has been studied extensively in neuromuscular populations [15,16,17] but also in stroke populations [18,19]. Prior studies in stroke suggest that reduced RSA reflects clinically meaningful limitations in paretic arm capacity [18], including impairments in motor control, strength, coordination, and independence with daily activities [19]. These findings indicate that RWS may capture stroke-related upper-extremity impairments in a spatially meaningful way, supporting its use as a quantitative measure of arm capacity during stroke rehabilitation. More recently, advances in markerless computer vision have expanded the technical approaches available for upper-extremity motion assessment. Portable markerless motion-capture systems have demonstrated validity and reliability for assessing upper-limb range of motion and joint kinematics during functional tasks [20]. Monocular AI-based motion capture has also recently been validated for quantifying upper-extremity reachable workspace, supporting the feasibility of lower-cost and more accessible camera configurations while identifying challenges related to depth estimation and posterior workspace measurement [21].

Nevertheless, a central challenge in stroke rehabilitation is that clinical capacity does not always translate into real-world performance. Many stroke survivors demonstrate some ability to move the affected arm and hand in structured clinical settings but use it substantially less during daily life [22]. This discrepancy is often described as a “capacity–performance” mismatch, where what a person can do under supervised or instructed conditions differs from what they actually do at home [23,24]. This framework provides important context for comparing clinic-based measures of motor capacity, self-reported arm use, and smartwatch-derived movement behavior. However, the present study was not designed to quantify this capacity–performance mismatch at the individual level.

To capture home use, wearable sensors are increasingly used to measure upper-extremity activity after stroke [25]. Widely available wrist-worn accelerometers and inertial measurement units (IMUs) can monitor movement continuously and unobtrusively during daily life. Prior studies have quantified relative real-world upper-limb use using bilateral accelerometry metrics such as the use ratio and magnitude ratio [26], activity counts [27], and hours of use [28], while newer approaches have explored kinematic and movement-quality features to characterize not only how much the arm moves, but also how it moves [29,30]. These tools show potential for home-based monitoring, where continued upper-extremity support is often needed after discharge [31], and consumer smartwatches may provide a scalable platform for feedback, goals, or reminders to encourage paretic arm use [32,33,34,35,36,37]. However, wearable accelerometry metrics focused on activity levels do not directly indicate how daily arm movement relates to specific arm impairments [22].

Therefore, while RWS may be especially useful in stroke rehabilitation as a quantitative and structured measure of upper-extremity “capacity” and recovery, wearable inertial sensors could provide information about how often, how intensely, and potentially how diversely the arm is used outside the clinic [23,25,29,30,32,38]. Wearable technologies may therefore provide insight into the real-world relevance of RWS by linking clinical movement “capacity” with everyday upper-extremity “performance”.

In this study, we used a commercially available smartwatch to compare camera-based reachable workspace outcomes with wearable accelerometry-derived upper-extremity movement metrics in individuals post-stroke. Specifically, we examined how global and quadrant-specific RSA measures relate to smartwatch-derived measures of arm movement. By linking RSA with wearable IMU data, this study aimed to determine whether accelerometry-based movement metrics were associated with upper-extremity reachable workspace and to explore whether the two approaches may capture related but distinct aspects of post-stroke arm function. To our knowledge, this is the first study to directly investigate the relationship between reachable workspace and wrist-worn IMU-based movement metrics in stroke.

## 2. Materials and Methods

### 2.1. Participants

Fourteen individuals with chronic stroke (>6 months post stroke event) were enrolled through a stroke survivor research database at the study institution and local stroke support groups. Demographic and baseline clinical characteristics are presented in Table 1. All study procedures were approved by the UC Irvine Institutional Review Board and participants provided informed consent. The study was registered on ClinicalTrials.gov under identifier NCT07042152.

### 2.2. Protocol

This study used baseline measurements collected as part of a 9-week clinical trial designed to examine the effects of smartwatch-based feedback on paretic-arm use after stroke. At the baseline visit, participants completed screening followed by the Box and Block Test (BBT), Upper-Extremity Fugl-Meyer Assessment (UEFM), Motor Activity Log Amount of Use and Quality of Movement scales (MAL-AOU and MAL-QOM), and reachable workspace (RWS) assessment, in that order. After completing these assessments, participants received a Samsung Galaxy Watch5 Pro, sampling at 50 Hz, with instructions for home use. Participants then wore the smartwatch during the following seven days, allowing habitual upper-extremity movement behavior to be characterized. Thus, all clinical and RWS assessments included in the present analysis preceded the collection of the corresponding free-living smartwatch data.

The baseline week was followed by four weeks of smartwatch feedback and a subsequent four-week retention period without feedback, with additional in-person assessments conducted after each phase. The present analysis included only the clinical and RWS assessments collected at the baseline visit and the smartwatch data collected during the immediately following no-feedback week. No data from the feedback or retention phases were included.

Participants were excluded if they were less than six months post-stroke; had substantial impairment in alertness, language reception, or attention; severe upper-extremity muscle tone; severe pain; advanced systemic disease; a coexisting major neurological or psychiatric condition; or another medical contraindication identified by the study physician. Participants were also excluded if they planned to alter their participation in concurrent rehabilitation therapy during the study or were enrolled in another study related to stroke recovery. The study did not direct changes to participants’ medication regimens.

The present study was designed as an exploratory analysis of the baseline data from the parent 9-week trial. Its purpose was to estimate preliminary associations between clinic-based reachable workspace and free-living wearable outcomes and to generate hypotheses for evaluation in larger cohorts, rather than to provide definitive population-level estimates.

### 2.3. Outcome Measures

#### 2.3.1. Reachable Workspace (RWS) and Relative Surface Area (RSA)

**Figure 1:**
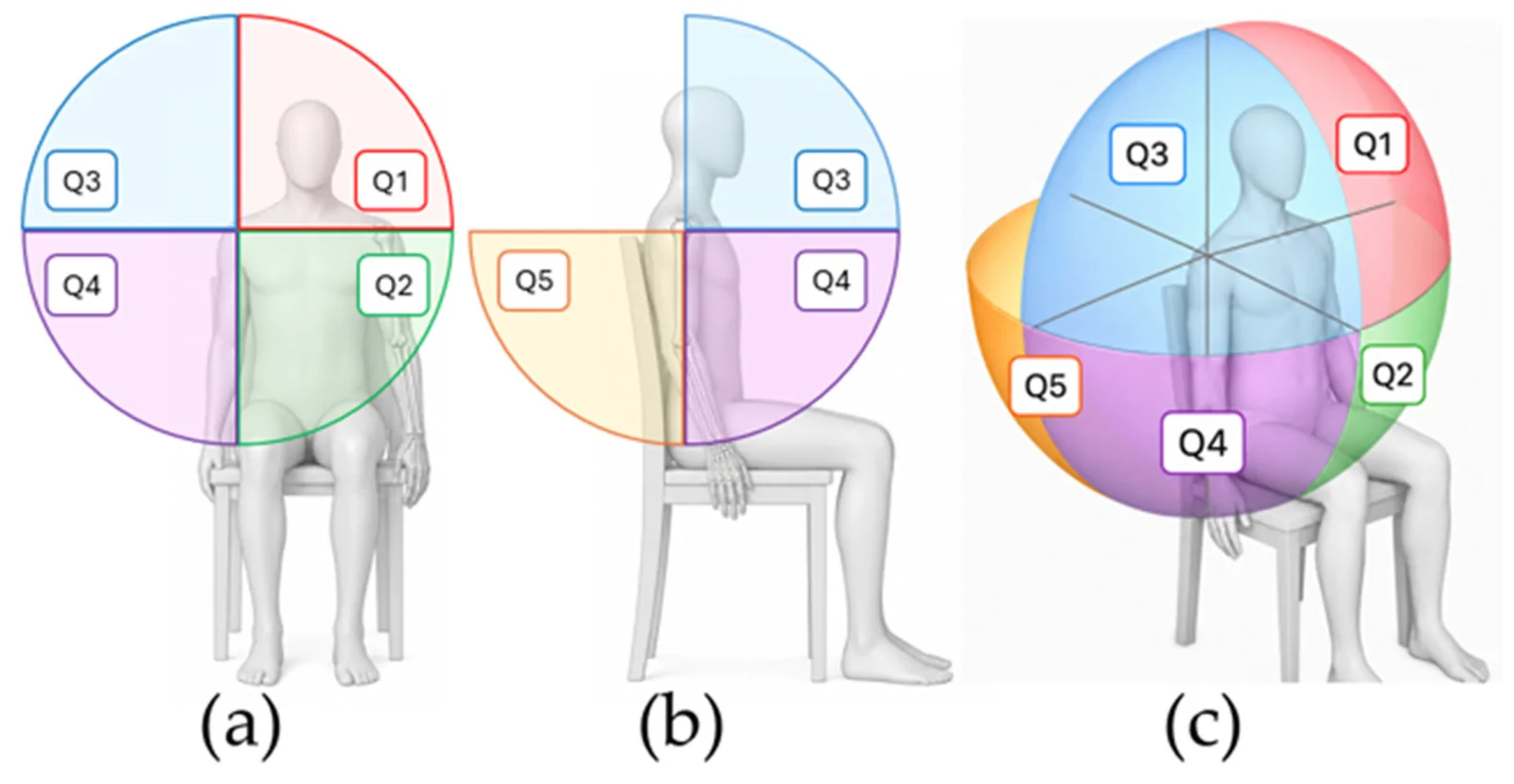
Reachable workspace quadrant definition. Three-dimensional shoulder-centered reachable workspace model used to quantify upper-limb reachability. The shoulder joint serves as the origin of the workspace, which is divided into four anterior quadrants and one posterior quadrant. Q1 and Q3 represent above-shoulder medial and lateral reach, respectively, while Q2 and Q4 represent below-shoulder medial and lateral reach. Q5 represents posterior lower-lateral reach behind the body. (**a**) Frontal view; (**b**) lateral view; (**c**) elevated anterolateral oblique view.

RWS was assessed using relative surface area (RSA) [12], a portable depth sensor-based, markerless motion-capture outcome that quantifies the three-dimensional space that each upper limb can actively reach (Figure 1). Reachable workspace was assessed using the previously published standardized protocol [12]. Briefly, wrist trajectories were mapped onto a shoulder-centered hemispherical model to calculate relative surface area (RSA), normalized to each participant’s arm length. Assessments were performed without added weight and with a 500 g wrist weight. The weighted condition was included as an exploratory test of reduced motor reserve under distal loading and was not intended to simulate smartwatch wear, as the smartwatch weighed approximately 50 g. RSA was reported as a total score and by anatomical quadrants (Figure 1), including upper-medial (Q1), lower-medial (Q2), upper-lateral (Q3), lower-lateral (Q4), and posterior lower-lateral regions (Q5), allowing assessment of both global and region-specific reaching limitations.

#### 2.3.2. Wearable Data (GMAC and ADEM)

**Figure 2:**
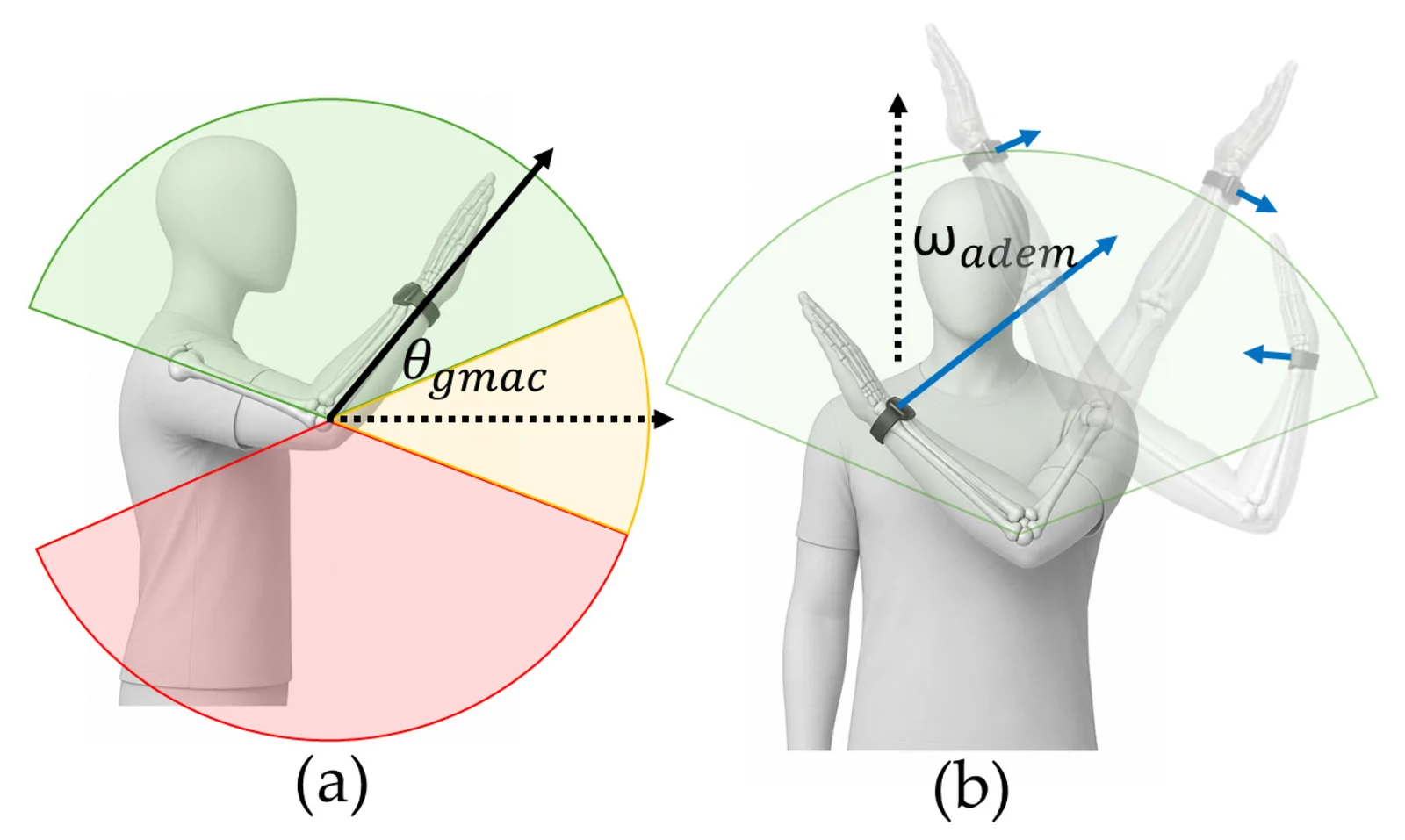
Wearable-derived movement metrics. (**a**) GMAC metric illustration [39]. Movements are classified as functional and counted only when the forearm is elevated within the predefined functional workspace (green), or when in the yellow region after coming from the green region, and the accelerometer magnitude exceeds a predefined activity threshold. Movements in the red region are never counted. The forearm inclination angle, θgmac, is used to determine whether movement occurs above the elevation threshold and therefore contributes to GMAC; (**b**) ADEM metric illustration. The ωadem angle represents changes in wrist orientation relative to the gravity vector during movement. Variability in this orientation angle (blue vector pointing out of the watch’s screen) is used to quantify active and diverse exploratory movement patterns within the same functional workspace as in GMAC.

Smartwatch-derived upper-extremity movement was summarized using two outcomes: GMAC-derived active movement ratio [39], and ADEM-derived diverse movement ratio [30,40,41]. Gross Movement and Activity Count (GMAC) quantifies active movement within a functional arm workspace by identifying periods when the arm exceeded predefined acceleration and forearm inclination thresholds (Figure 2a). Active and Diverse Exploratory Movement (ADEM) quantifies movement that is both active and diverse by identifying active periods in which the distribution of wrist orientation over the preceding minute showed sufficient variability, reflected by kurtosis [30] below a predefined threshold (Figure 2b). For each day, active time was computed as the total duration for which the GMAC activity flag was positive, and diverse time was computed as the total duration for which the ADEM diversity flag was positive.

Wearable data were analyzed over each participant’s first seven baseline days. A valid day required at least 480 min of smartwatch wear time and finite daily GMAC and ADEM ratios. Days were excluded if wear time was below 480 min, wear-time data or daily movement metrics were unavailable, or the resulting GMAC or ADEM ratio was nonfinite. Daily GMAC and ADEM values were normalized by dividing the corresponding movement duration by daily smartwatch wear time and were expressed as percentages of wear time. This normalization reduced the influence of differences in daily recording duration across participants and days. Participant-level baseline estimates were obtained from the available valid days and used as the wearable-derived measures of real-world upper-extremity movement behavior. Full details of the sensing platform, real-time implementation, signal processing, and algorithm thresholds are provided elsewhere [40].

#### 2.3.3. Upper-Extremity Clinical and Self-Reported Assessments (BBT, UEFM, and MAL)

Upper-extremity motor impairment and function were characterized using the Upper-Extremity Fugl-Meyer Assessment (UEFM) [7] and the Box and Block Test (BBT) [42]. The UEFM is a standardized, performance-based measure of post-stroke upper-extremity motor impairment, with higher scores indicating less impairment. The motor section ranges from 0 to 66 and evaluates movement, coordination, and reflex activity across the shoulder, elbow, forearm, wrist, and hand. The BBT is another widely used assessment to assess gross manual dexterity after stroke [42]. Participants are instructed to transfer as many wooden blocks as possible, one at a time, over a partition during a 60 s period using the paretic hand. The score is the total number of blocks successfully transferred, with higher scores indicating better gross manual dexterity.

Self-reported real-world use of the paretic upper extremity was assessed using the 14-item Motor Activity Log (MAL-14) [43]. The MAL-14 is a structured interview in which participants rate how much and how well they used the paretic arm during common daily activities. It produces two mean scores ranging from 0 to 5: the Amount of Use scale (MAL-AOU), reflecting how frequently the arm was used, and the Quality of Movement scale (MAL-QOM), reflecting the participant’s perceived quality of movement during arm use. Higher scores indicate greater use and better perceived movement quality. The MAL-14 was included as a patient-reported outcome (PRO) measure of real-world upper-extremity performance, supplementing the clinic-based measures of impairment and capacity.

### 2.4. Statistics and Data Analysis

#### 2.4.1. Associations Between RSA and Wearable Data, Upper-Extremity Clinical Outcomes, and Self-Reported Assessments

Associations between baseline paretic-arm Total RSA and the six clinical, self-reported, and smartwatch-derived outcomes (Table 2), comprising BBT, UEFM, MAL-AOU, MAL-QOM, GMAC, and ADEM, were assessed using two-sided Spearman rank correlations. This rank-based approach was selected because several outcomes were ordinal or bounded and were not assumed to follow normal distributions. Spearman’s ρ was reported as the standardized effect-size estimate for each association, describing its magnitude and direction. Given the small sample, the precision of each effect-size estimate was quantified using participant-level 95% bias-corrected and accelerated (BCa) bootstrap confidence intervals based on 10,000 paired resamples. Within each resample, the RSA and corresponding outcome observations were resampled together at the participant level. These confidence intervals were calculated separately for each association and describe the uncertainty surrounding its estimated Spearman’s ρ. Conventional two-sided raw *p*-values were obtained for each Spearman correlation. Because the six Total RSA associations addressed the same overall research question, they were defined as one family of statistical tests. To account for multiple comparisons, the six raw *p*-values were adjusted together using the Benjamini–Hochberg procedure, which controls the expected proportion of false discoveries among findings declared statistically significant. Thus, the confidence intervals were used to characterize the precision of each individual effect-size estimate, whereas multiplicity was addressed by adjusting the six *p*-values. Both raw and BH-adjusted *p*-values are reported, with BH-adjusted *p* < 0.05 used as the criterion for statistical significance. The magnitude of Spearman’s ρ, its 95% confidence interval, and its BH-adjusted *p*-value were considered together when interpreting each association.

#### 2.4.2. Comparisons of Correlation Strengths

After estimating the individual RSA associations, three specified pairs of correlation coefficients were formally compared: BBT versus UEFM, MAL-AOU versus MAL-QOM, and ADEM versus GMAC. Within each comparison, the correlations were dependent and overlapping because they shared Total RSA and were calculated from the same participants; therefore, their numerical coefficients or individual *p*-values could not be compared directly. Because Spearman correlations are equivalent to Pearson correlations calculated on ranked observations, Total RSA and the corresponding outcomes were rank-transformed and compared using two-sided Williams tests, which account for the correlation between the two outcomes. Differences were expressed as Δρ, defined as the first RSA correlation minus the second. Participant-level 95% BCa bootstrap confidence intervals for Δρ were calculated using 10,000 paired resamples that preserved complete participant observations. The Williams tests provided the primary statistical inference, while the bootstrap intervals quantified uncertainty in Δρ. The three Williams-test *p*-values were adjusted together using the Benjamini–Hochberg procedure as a separate multiple-comparison family, with BH-adjusted *p* < 0.05 indicating a statistically significant difference in correlation strength.

#### 2.4.3. Exploratory Region-Specific Associations and Comparisons of Dependent Correlation Strengths Across Quadrants and Composite Regions

All region-specific analyses were considered exploratory because of the increased number of correlated tests and the spatial overlap among the individual quadrants and composite RSA measures. The region-specific analyses extended the frameworks defined in Section 2.4.1 and Section 2.4.2 to a finer spatial resolution of RSA. Specifically, the five individual quadrants (Q1–Q5) and four composite regions (upper, lower, medial, and lateral RSA) were analyzed. For the association analysis, each of the nine RSA measures was evaluated against the six clinical, self-reported, and smartwatch-derived outcomes described in Section 2.3. This resulted in 54 region-specific Spearman correlations. The corresponding correlation coefficients, bootstrap confidence intervals, and Benjamini–Hochberg-adjusted *p*-values were calculated using the procedures defined in Section 2.4.1, with the 54 tests treated as a separate regional association family. For the dependent correlation analysis, the procedure described in Section 2.4.2 was applied separately within each of the nine RSA measures. For example, within Q1, the correlation between Q1 RSA and ADEM was formally compared with the correlation between Q1 RSA and GMAC. The corresponding BBT-versus-UEFM and MAL-AOU-versus-MAL-QOM comparisons were performed in the same manner. Repeating the three planned outcome-pair comparisons across the nine RSA measures yielded 27 region-specific comparisons of dependent correlations. For each comparison, the Williams test and bootstrap-based estimation of Δρ were performed as defined in Section 2.4.2. The resulting 27 *p*-values were adjusted together using the Benjamini–Hochberg procedure as a separate regional comparison family.

#### 2.4.4. Outlier Removal

Outliers were identified using a median absolute deviation (MAD)-based modified z-score threshold of |z| > 3.5. Identified outliers were marked with crosses in the figures for transparency. Outliers were retained in the primary correlation analyses unless the plot was specifically configured as an outlier-exclusion sensitivity analysis.

#### 2.4.5. Plotting Statistical Concepts

**Figure 3:**
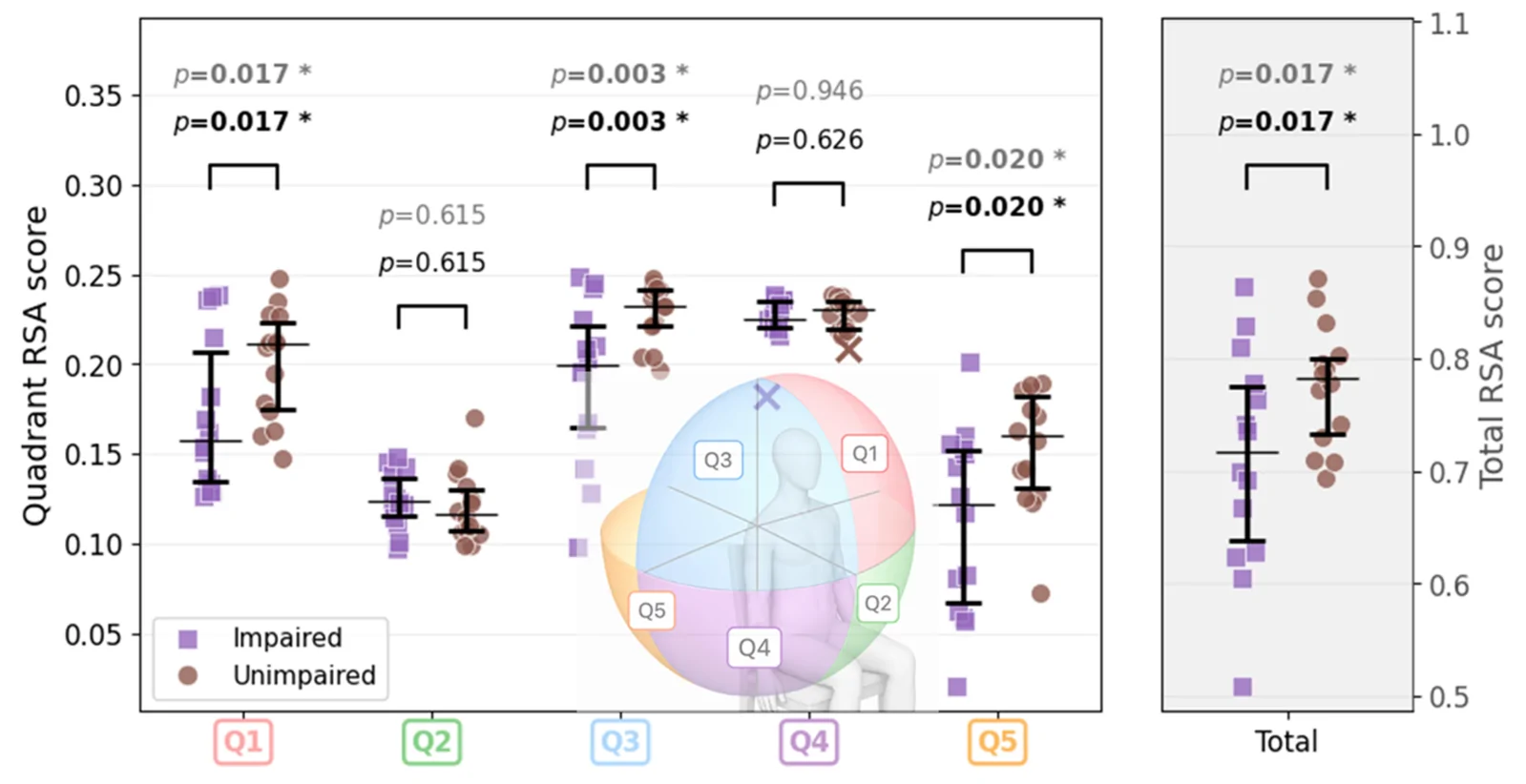
Baseline RWS distinguishes impaired vs. unimpaired upper limbs. Baseline RSA values are shown for each RWS quadrant and for the “Total” score adding all quadrants. Purple squares represent the impaired limb, and brown circles represent the unimpaired limb. Individual points show participant-level values, black horizontal lines indicate group medians, and error bars indicate variability using IQR quartiles. Bold *p*-values indicate the primary paired Wilcoxon test using all paired observations, whereas gray *p*-values indicate the sensitivity analysis after excluding MAD-identified outliers. * denotes significance. Q1 to Q5 scores ranging from 0 to 0.25 are plotted on the left y-axis, and total RSA score with a maximum of 1.25 is plotted on the right y-axis.

**Figure A1:**
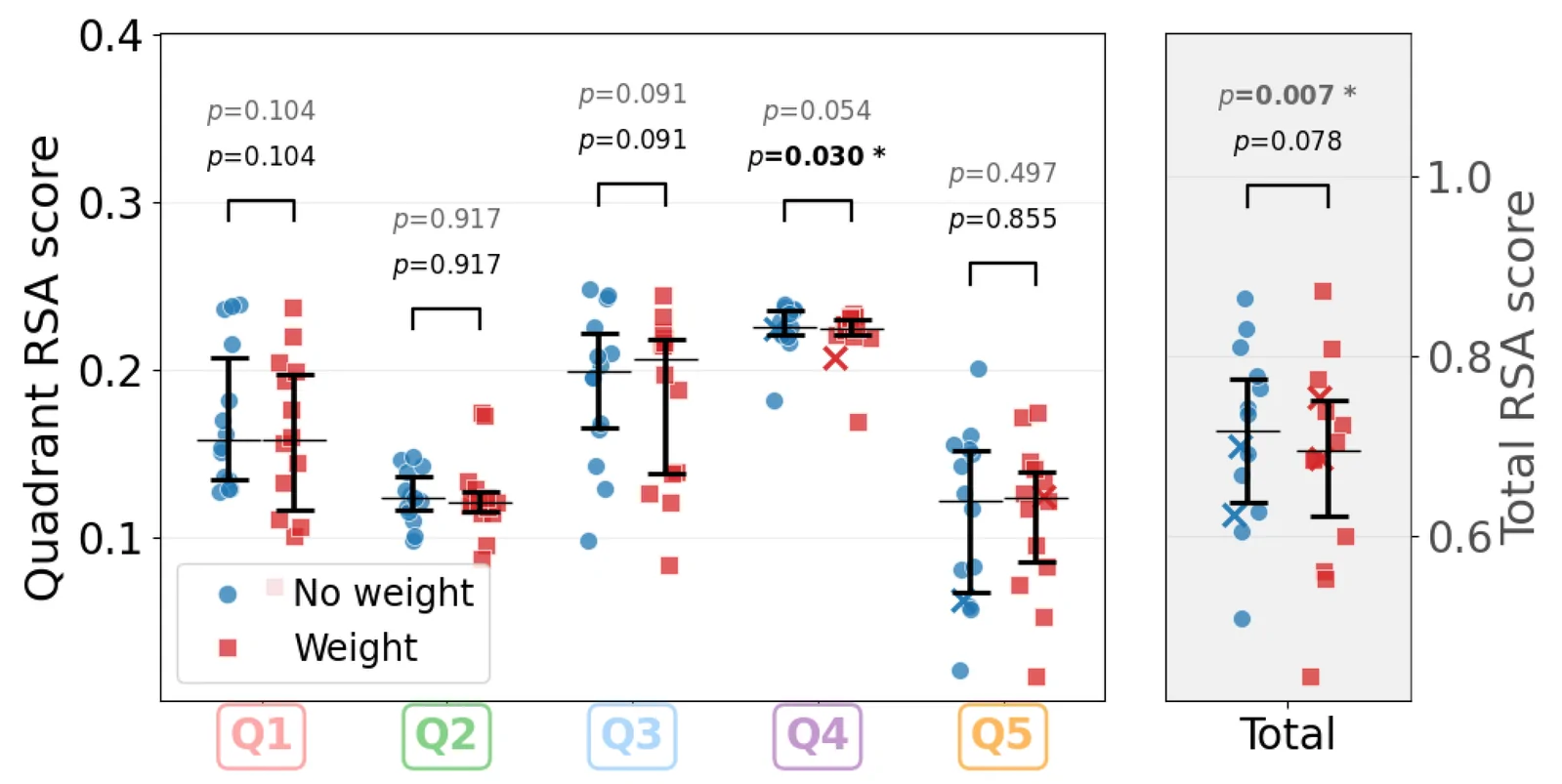
Effect of added weight on reachable workspace RSA by quadrant and total score. Individual participant RSA scores are shown for no-weight trials (blue circles) and weighted trials (red squares), with horizontal bars indicating the group median and vertical bars indicating the interquartile range. Cross markers denote paired-difference outliers identified using a MAD-based robust z-score. * denotes significance. Quadrant RSA scores are plotted on the left y-axis, while total RSA is plotted on the right y-axis and separated by the shaded region. Brackets show paired Wilcoxon signed-rank comparisons between no-weight and weight conditions. Black *p*-values indicate the primary analysis using all paired observations, and gray *p*-values indicate the sensitivity analysis after removing paired-difference outliers.

Consistent with the use of Spearman rank correlations, Figure 3 and Figure A1 present rank-based, non-parametric summaries rather than mean-based summaries. Central tendency is represented by the median, which is less sensitive to extreme values and is consistent with the non-parametric and outlier-robust analytical approach. Variability is represented using the interquartile range (IQR), extending from the 25th percentile (Q1) to the 75th percentile (Q3), with the median indicated at the center. Paired Wilcoxon signed-rank tests were used to compare impaired and unimpaired arm’s RSA scores within each quadrant (Figure 3).

Best-fit curves displayed in the scatterplots were selected from candidate linear, quadratic, square-root, exponential, logarithmic, and saturating models based on the lowest Akaike Information Criterion (AIC).

## 3. Results

### 3.1. Participant Characteristics and Baseline Clinical Status

Fourteen individuals in the chronic phase post-stroke participated in the study. Participants were 60.0 ± 11.6 years old, the cohort included 3 females and 11 males, and the mean time since stroke was 6.3 ± 2.9 years. Additional demographic and baseline clinical characteristics are presented in Table 1. At the baseline appointment, participants completed a battery of assessments to characterize demographics, clinical motor capacity and impairment, and self-reported arm use. Participants then wore the smartwatch for one week to characterize baseline wearable-derived movement behavior during daily life. Mean daily smartwatch wear time was 12.13 ± 0.72 h; the mean number of days for which participants wore the watch for at least 8 h was 6.64 ± 0.63, ranging from five to seven days. Five of the 98 potential participant-days were excluded because wear time was below 480 min. These measures are summarized in Table 1 and organized by assessment domain.

**Table 1:** Demographic and baseline clinical characteristics of the study participants (mean ± SD).

Demographics (N = 14)	
Age	60 ± 11.6 years
Sex Assigned at Birth	3 females; 11 males
Time Since Stroke	6.3 ± 2.9 years
Paretic Side	8 right; 6 left
Hand Dominance	12 right; 2 left
Stroke Type	5 hemorrhagic; 7 ischemic; 2 unknown
Clinical motor capacity/impairment	
Box and Block Test (BBT)	31.6 ± 12.8 blocks
Upper-Extremity Fugl-Meyer Assessment (UEFM)	52.7 ± 7.3 (maximum of 66)
Reachable Workspace (total RSA including Q5)	0.71 ± 0.09 (maximum of 1.25)
Self-reported behavior/use	
Motor Activity Log Amount Of Use (MAL-AOU)	2.02 ± 0.81 (from 0 to 5)
Motor Activity Log Quality Of Movement (MAL-QOM)	1.99 ± 0.85 (from 0 to 5)
Wearable-derived measures	
GMAC	21.5 ± 13.9% of smartwatch wear time
ADEM	9.7 ± 9.3% of smartwatch wear time

### 3.2. Baseline RWS Demonstrates Impaired-Limb Reachability Deficits and Limited Sensitivity to Added Load

Baseline RSA showed clear differences between the impaired and unimpaired upper limbs (Figure 3). RSA was lower in the impaired limb for Q1, Q3, Q5, and total RSA, indicating reduced reachable workspace in the affected arm. The largest quadrant-level differences were observed in Q3 and Q5, while Q2 and Q4 (lower quadrants) did not show significant between-limb differences. Total RSA was also significantly lower in the impaired limb, supporting the sensitivity of RWS for detecting quadrant-specific capacity differences after stroke. The 500 g wrist-weight condition produced limited quadrant-level effects (Figure A1) and therefore subsequent clinical and wearable association analyses focused only on the no-weight RSA condition.

### 3.3. Reachable Workspace Is Associated with Clinical, Self-Reported, and Smartwatch-Derived Outcomes

Total RSA showed positive correlations with all six clinical, self-reported, and smartwatch-derived outcomes, although the magnitude and uncertainty of these associations varied (Table 2). The strongest association was observed for MAL-AOU (ρ = 0.76, 95% CI [0.43, 0.95], BH-adjusted *p* = 0.009). Total RSA was also associated with BBT (ρ = 0.69, 95% CI [0.47, 0.91], BH-adjusted *p* = 0.014), UEFM (ρ = 0.68, 95% CI [0.19, 0.90], BH-adjusted *p* = 0.014), ADEM (ρ = 0.66, 95% CI [0.05, 0.90], BH-adjusted *p* = 0.015), and MAL-QOM (ρ = 0.57, 95% CI [−0.14, 0.88], BH-adjusted *p* = 0.040). Although the BH-adjusted *p*-value for MAL-QOM was below 0.05, its confidence interval included zero, indicating substantial uncertainty in the estimated association. The association with GMAC was numerically smaller and did not remain statistically significant (ρ = 0.47, 95% CI [−0.21, 0.79], BH-adjusted *p* = 0.091). Region-specific associations are examined in the following sections.

**Table 2:** Associations Between Total Relative Surface Area and Clinical, Self-Reported, and Smartwatch-Derived Outcomes.

Outcome	Spearman ρ	95% CI	Raw *p*	BH-Adjusted *p*
BBT	0.69	[0.47, 0.91]	0.007	0.014
UEFM	0.68	[0.19, 0.90]	0.007	0.014
MAL-AOU	0.76	[0.43, 0.95]	0.002	0.009
MAL-QOM	0.57	[−0.14, 0.88]	0.033	0.040
GMAC	0.47	[−0.21, 0.79]	0.091	0.091
ADEM	0.66	[0.05, 0.90]	0.010	0.015

**Figure 4:**
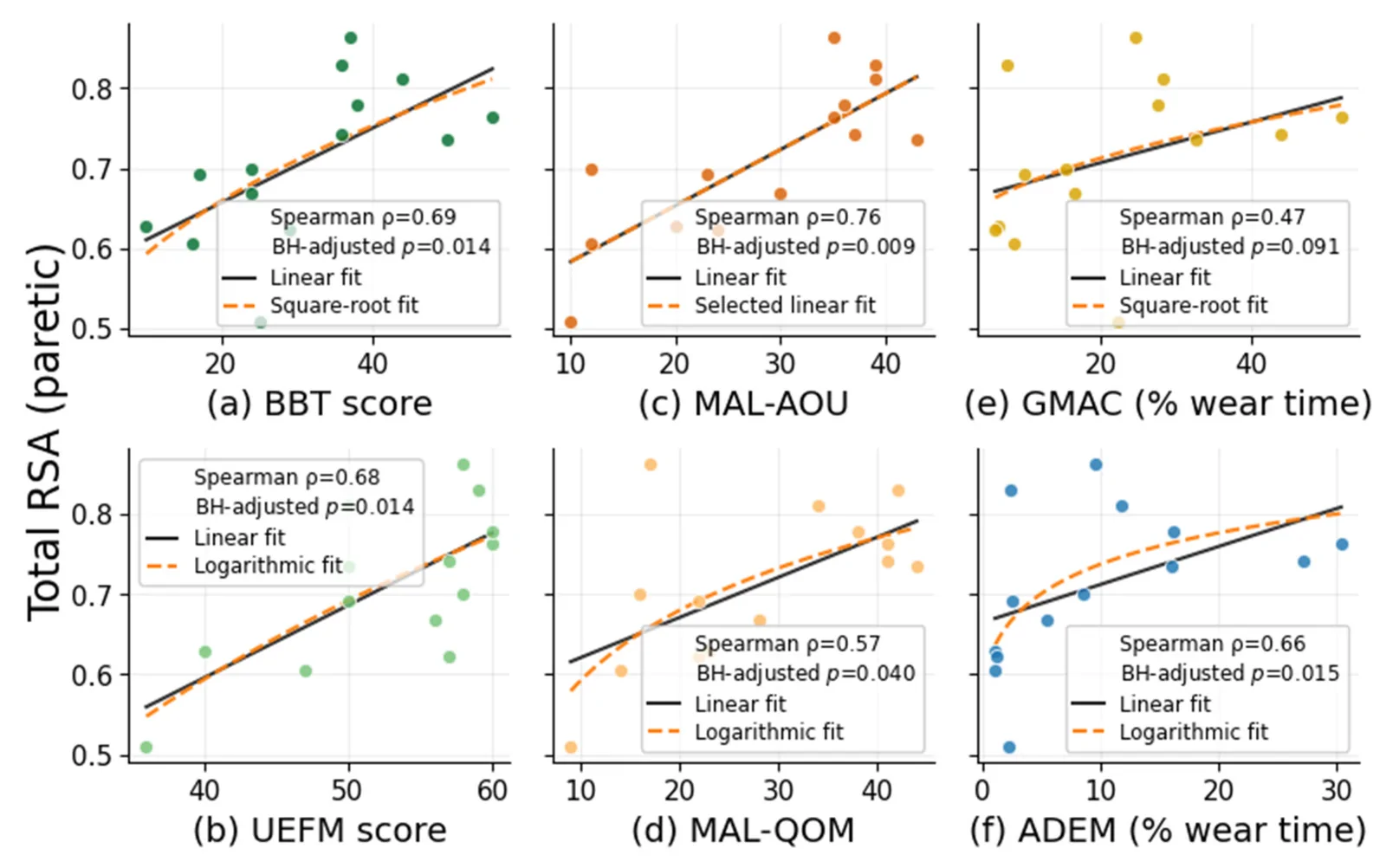
Participant-level associations between Total Relative Surface Area and clinical, self-reported, and smartwatch-derived outcomes. Baseline paretic-arm Total RSA is plotted against (**a**) BBT, (**b**) UEFM, (**c**) MAL-AOU, (**d**) MAL-QOM, (**e**) GMAC, and (**f**) ADEM in 14 participants. GMAC and ADEM represent the percentage of smartwatch wear time meeting each movement criterion during the no-feedback baseline week. Each point represents one participant. Solid black lines represent linear fits, and orange dashed lines represent the selected nonlinear best fits. These curves are provided for descriptive visualization only; statistical inference was based on Spearman rank correlations reported in Table 2.

Participant-level scatterplots of the six associations are presented in Figure 4. Linear and selected nonlinear fits are included for descriptive visualization, whereas statistical inference was based on the Spearman rank correlations reported in Table 2. Region-specific associations are examined in the following sections.

### 3.4. Reachable Workspace Correlation Strengths Did Not Differ Significantly Between Paired Outcomes

None of the three dependent-correlation comparisons demonstrated a statistically significant difference in correlation strength after Benjamini–Hochberg correction (Table 3). The associations of Total RSA with BBT and UEFM were nearly identical (ρ = 0.69 and ρ = 0.68, respectively; Δρ = 0.001, 95% BCa CI [−0.32, 0.52]), with no evidence of a difference between them, Williams t(11) = 0.003, BH-adjusted *p* = 0.997. Total RSA was numerically more strongly associated with MAL-AOU than with MAL-QOM (ρ = 0.76 versus ρ = 0.57; Δρ = 0.19, 95% BCa CI [−0.02, 0.86]), but this difference only approached significance, Williams t(11) = 2.13, BH-adjusted *p* = 0.086. Similarly, the association between Total RSA and ADEM was numerically larger than that between Total RSA and GMAC (ρ = 0.66 versus ρ = 0.47; Δρ = 0.19, 95% BCa CI [0.01, 0.78]), but the Williams test did not demonstrate a statistically significant difference, t(11) = 2.12, BH-adjusted *p* = 0.086. Although the bootstrap confidence interval for the ADEM-versus-GMAC difference excluded zero, this finding was not concordant with the multiplicity-adjusted Williams test. Therefore, the evidence was considered inconclusive, and no definitive difference in correlation strength was inferred.

### 3.5. Exploratory Region-Specific Associations Between Reachable Workspace and Study Outcomes

**Figure 5:**
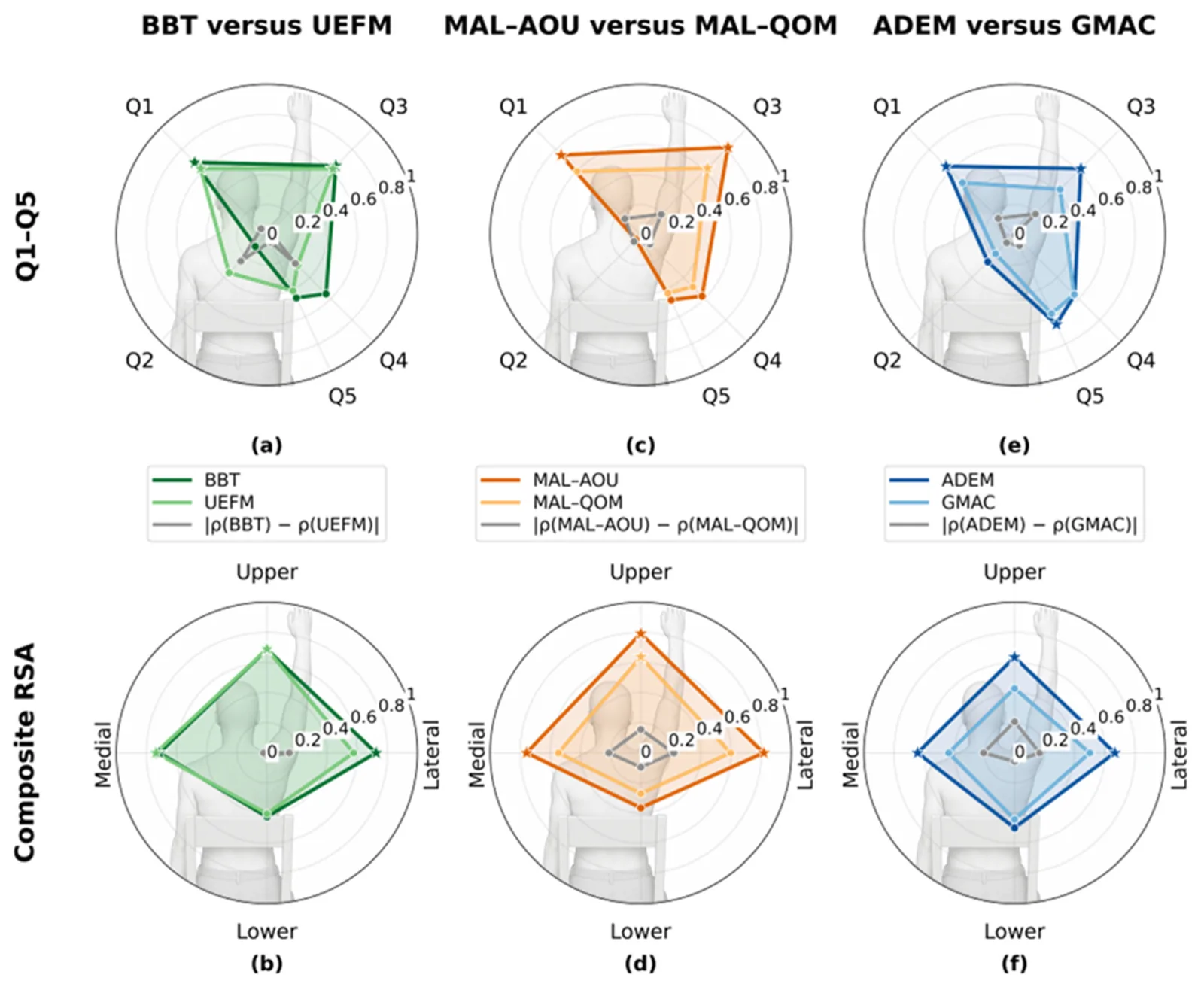
Region-specific associations between reachable workspace and clinical, self-reported, and wearable-derived outcomes. Columns compare BBT with UEFM, MAL-AOU with MAL-QOM, and ADEM with GMAC. The top row shows Spearman correlations for Q1–Q5, and the bottom row shows upper (Q1 + Q3), lower (Q2 + Q4), medial (Q1 + Q2), and lateral (Q3 + Q4) RSA. Colored lines represent the correlations between each RSA region and the corresponding outcome, while gray lines represent the absolute difference between the paired correlations, |Δρ| = |ρ_1_ − ρ_2_|. Colored stars indicate associations with BH-adjusted *p* < 0.05 across the 54 regional correlations; circles indicate adjusted *p* ≥ 0.05. Gray stars would indicate significant differences between paired correlations after BH correction across the 27 Williams tests; none were observed. (**a**) Q1–Q5, BBT versus UEFM; (**b**) composite RSA, BBT versus UEFM; (**c**) Q1–Q5, MAL–AOU versus MAL–QOM; (**d**) composite RSA, MAL–AOU versus MAL–QOM; (**e**) Q1–Q5, ADEM versus GMAC; (**f**) composite RSA, ADEM versus GMAC.

For each outcome (Section 2.3.2 and Section 2.3.3), Reachable Workspace was subdivided into five angular quadrants (Figure 5; row 1; Q1–Q5) representing directional sectors of the reachable workspace, and into four composite regions (Figure 5; row 2; upper, lower, medial, and lateral) that summarize broader anatomical directions. These subdivisions are illustrated in the spider plot (Figure 5), which visually maps the pattern and magnitude of correlations across all regions. Within each of the five RSA quadrants and four composite regions, correlation strength was compared for three outcome pairs (Figure 5; grey lines): BBT versus UEFM, MAL-AOU versus MAL-QOM, and ADEM versus GMAC. Across all comparisons, none of the differences was statistically significant. Therefore, no measure within any pair demonstrated a statistically stronger association with a specific RSA quadrant or composite region than its paired measure.

BBT showed its strongest numerical associations with Q1 (ρ = 0.68) among the quadrants and with medial and lateral RSA (both ρ = 0.72) among the composite regions. Its associations with Q1, Q3, upper, medial, and lateral RSA remained significant after correction. UEFM showed its strongest correlations with Q1 (ρ = 0.62) and medial RSA (ρ = 0.74), with significant associations persisting for Q1, Q3, upper, and medial RSA. None of the nine BBT-versus-UEFM comparisons demonstrated a statistically significant difference in correlation strength after BH correction.

MAL-AOU showed its strongest correlations with Q3 and lateral RSA (both ρ = 0.82), with significant associations for Q1, Q3, upper, medial, and lateral RSA. MAL-QOM showed its strongest correlations with Q3 (ρ = 0.63) and upper RSA (ρ = 0.64), both of which remained significant. None of the MAL-AOU versus MAL-QOM regional differences were significant after correction. For medial RSA, the BCa confidence interval for Δρ excluded zero (95% CI, 0.002 to 0.896), although the corresponding Williams test was not significant after correction (q = 0.251).

ADEM showed its strongest correlations with Q1 (ρ = 0.64) and lateral RSA (ρ = 0.67), with significant associations for Q1, Q3, Q5, upper, medial, and lateral RSA. GMAC showed its strongest correlations with Q4 (ρ = 0.56) and lateral RSA (ρ = 0.50), but none of its regional associations remained significant after correction. None of the ADEM versus GMAC regional differences were significant after correction. BCa confidence intervals for Δρ excluded zero for Q3 (95% CI, 0.01 to 0.83), upper RSA (0.03 to 0.77), and medial RSA (0.04 to 0.72), although the corresponding Williams tests were not significant after correction (Q3: q = 0.286; upper and medial RSA: q = 0.251).

## 4. Discussion

This study examined the relationship between reachable workspace using the camera-based RSA metric, and clinically assessed motor capacity measures, self-reported arm use, and smartwatch-derived real-world upper-limb performance in individuals with chronic stroke. Four main findings emerged: (1) baseline RWS identified clear impaired-limb reachability deficits while showing limited sensitivity to added distal load in the study’s stroke cohort; (2) greater RSA was associated with better clinical motor capacity, gross manual dexterity, and greater self-reported amount and quality of paretic-arm use; (3) RWS was associated with wearable-derived movement behavior for the first time, with movement diversity showing numerically stronger relationships than amount of active movement alone, although not significantly; and (4) quadrant-specific RSA revealed that upper, lateral, and posterior workspace regions were particularly informative for understanding clinical function and real-world arm use, providing meaningful information beyond total RSA.

### 4.1. Clinical Sensitivity of Reachable Workspace to Post-Stroke Upper-Limb Impairment

Baseline RWS was significantly lower for the impaired than the unimpaired limb in total RSA and several workspace regions, consistent with prior evidence that RSA sensitively captures clinically meaningful upper-limb reachability deficits after stroke [12], as well as being consistent with studies using other metrics of arm workspace after stroke [44,45,46,47,48]. Although the unaffected limb may also exhibit subtle post-stroke impairments [49,50] and should not be considered a truly normal control, the within-participant comparison confirmed that RWS distinguished the paretic limb in the present cohort. Subsequent clinical and wearable association analyses therefore focused on the impaired limb, since the smartwatch was worn on the affected wrist and the reported clinical outcomes were also assessed for that side. Analyses were further restricted to the no-weight condition because the 500 g load produced limited and outlier-sensitive effects across individual quadrants, supporting the decision to focus the primary analyses on the unloaded condition RWS. Together, these results reaffirm RWS as a clinically sensitive measure and support the use of impaired-limb, no-weight RSA for the primary analyses.

### 4.2. Reachable Workspace Is Associated with Clinical Capacity and Self-Reported Arm Use Quantity and Quality

Our findings align with prior Kinect-based RWS studies showing that RSA reflects clinician-rated upper-limb motor impairment after stroke. In the present study, total RSA was associated with UEFM scores, with a correlation magnitude similar to that previously reported by Lee et al. [18], who found that paretic-side RSA correlated with UEFM and UE Motricity Index scores in individuals with hemiplegic stroke. Similarly, Okuyama et al. [51] reported a strong correlation between the RWS ratio of the paretic and nonparetic upper limbs and UEFM scores, along with good reliability and high responsiveness after training. This consistency with prior work supports RWS as a valid and objective measure of upper-limb impairment and reachable workspace limitations.

In addition, we observed a significant association between total RSA and BBT performance. To our knowledge, this relationship has not been previously reported. This finding is clinically plausible because successful daily arm use requires not only the ability to move the arm through space, but also sufficient hand function to grasp, transport, and release objects. Furthermore, although not always, a typical pattern of stroke motor recovery follows proximal to distal progression [52,53], where by the time distal hand function demonstrates functional recovery, proximal shoulder and upper arm function has also been achieved. In other cases, even when proximal reaching capacity is present, limited hand recovery may reduce the practical usefulness of the affected arm during daily activities, decreasing both the opportunity and motivation to use it. The observed association between RSA and BBT suggests that individuals with greater reachable workspace may also have better gross manual dexterity, supporting a closer link between RWS capacity and functional arm use.

After establishing the relationship between RWS and clinician-administered motor outcomes, our findings further suggest that RSA is related to self-assessed daily functional arm use quantity and quality, measured using MAL. Previous work showed that sensor-acquired RSA was associated with clinician-evaluated FIM Self-Care after stroke, supporting the interpretation that reachable workspace may reflect the movement capacity needed to perform meaningful daily activities [19]. Importantly, Chan et al. [19] also reported strong associations between RSA and Neuro-QoL Upper-Extremity Function. Similarly, Lee et al. [18] found that paretic-side RSA was associated with QuickDASH, further linking RWS with patient-perceived upper-limb disability. In the present study, we extend these findings by showing that total RSA was associated with both MAL Amount of Use and MAL Quality of Movement. To our knowledge, the relationship between RWS and MAL has not been previously reported. This association is clinically meaningful because MAL directly captures how much and how well participants perceive they use the paretic arm during everyday activities. Participants with larger reachable workspaces may have greater ability to position the arm in functional regions of space, making the impaired limb more useful during daily tasks and increasing perceived amount and quality of use. Conversely, participants with smaller reachable workspaces may experience greater difficulty incorporating the impaired arm into daily activities, which may be reflected in lower MAL-AOU and MAL-QOM scores.

### 4.3. Reachable Workspace Is Associated with Wearable Metrics of Arm Activity

Although clinical assessments and patient-reported outcomes provide clinically meaningful information about upper-limb impairment, capacity, and perceived function, they do not directly quantify how much the affected arm is actually used during daily life. This distinction is important because real-world hand and arm use is ultimately what matters most to individuals after stroke. Wearable sensors offer a way to measure this everyday performance outside the clinic, but prior work relating wearable-derived upper-limb activity to clinical and self-reported outcomes has been mixed, with some studies reporting meaningful associations while others showing weaker or inconsistent relationships. For example, Bhatnagar et al. [28] reported that accelerometry-derived upper-limb activity metrics, particularly mean magnitude ratio, were associated with FMA, WMFT, ARAT, and MAL scores, and Kools et al. reported associations between wearable-derived functional performance variables and clinical outcomes in FSHD [54]. However, Hayward et al. [55] noted that most accelerometers are unable to distinguish the type or quality of movements performed, which may limit the clinical interpretation of raw activity-based metrics despite their potential to provide meaningful information for clinicians, patients, and caregivers. Consistent with this limitation, Pohl et al. [56] emphasized that real-world wrist-sensor data contain a mixture of functional arm movements and non-functional movement, and that conventional activity-count thresholds may overestimate functional activity because of low specificity.

Differences in how wearable metrics define and quantify arm use may therefore help explain the variability in their reported relationships with clinical outcomes. In the present study, GMAC and ADEM were designed to move beyond raw movement quantity by identifying activity within a functional arm workspace. GMAC quantifies active movement that occurs above a predefined forearm elevation threshold, while ADEM further identifies active movement accompanied by variability in forearm orientation, thereby capturing a more diverse repertoire of movement through three-dimensional space. These features may make GMAC and ADEM more clinically interpretable than conventional activity counts, because they are intended to reflect how the arm is used within functionally relevant spatial regions rather than simply whether movement occurred. RWS provides a particularly appropriate clinical measure for evaluating these wearable outcomes because RSA quantifies the normalized three-dimensional space that an individual can actively reach during a structured assessment. In this sense, RWS characterizes clinic-based spatial capacity, while GMAC and ADEM characterize how movement within a related functional space is expressed during daily life.

To our knowledge, no prior study has directly compared reachable workspace with wrist-worn accelerometry metrics in persons post-stroke. Total RSA was positively associated with both wearable outcomes, although the association was numerically larger for ADEM than for GMAC. This difference was not statistically significant in the formal dependent-correlation comparison and should therefore not be interpreted as evidence that ADEM is more strongly associated with RSA than GMAC. The numerically weaker GMAC association may reflect that the amount of active movement performed during daily life depends not only on an individual’s motor capacity, but also on opportunities for arm use, daily routines, motivation, compensatory strategies, and behavioral choices. ADEM incorporates movement diversity and may therefore capture a different aspect of how spatial reaching capacity is expressed during daily life. These findings are consistent with the capacity–performance mismatch framework by demonstrating that clinic-based spatial capacity and free-living movement behavior are related but not equivalent constructs. However, correlations at the group level do not identify whether individual participants use their paretic arm less than expected given their available capacity. How best to quantify the capacity–performance mismatch, is still an open question, with many studies relying on ordinal clinical measures of capacity and self-reported ratings of performance. Future studies should directly quantify participant-level capacity–performance mismatch and investigate the clinical and behavioral factors contributing to it.

### 4.4. Clinical and Real-World Relevance of Quadrant-Specific RSA

Beyond total RSA, quadrant-specific analyses may help clarify which regions of reachable workspace are most closely related to clinical function and real-world arm use. Previous studies demonstrate that post-stroke reaching deficits are not spatially uniform, with movement quality, coordination, and compensatory strategies varying according to target direction and workspace location [18,19,57,58,59]. In a previous three-dimensional RWS study, paretic RSA was significantly reduced in Q1, Q3, and Q4, and Q3 was the strongest regional determinant of upper-extremity impairment and disability [18,19]. Direction-dependent disturbances in paretic reaching have also been demonstrated using multidirectional reaching paradigms [58,59].

In the present study, Q1 and Q3 and the corresponding upper region showed some of the strongest associations with clinical motor outcomes, self-reported arm use, and wearable-derived movement. One possible explanation is that reaching into these regions places greater demands on active arm elevation and multi-joint shoulder-elbow coordination. Following stroke, increasing shoulder-abduction demand can amplify abnormal coupling between shoulder abduction and elbow flexion and progressively restrict the available reaching work area [57]. In contrast, the generally weaker relationships observed for Q2 and the lower region may reflect the availability of near-body or compensatory strategies for accessing portions of the lower workspace. This does not imply that lower-medial reach is clinically unimportant, since prior work has linked Q2, Q4, and Q5 with self-care activities such as dressing, toileting, and bathing [19]. These regional patterns should be interpreted cautiously because reaching impairment after stroke is heterogeneous and previous work has also demonstrated substantial deficits outside the upper workspace [44].

Q5 may provide complementary functional information because it represents posterior inferior-lateral reach, a region not well characterized by conventional forward-reaching assessments. Exploring this space is required for activities such as reaching behind the body, dressing, and toileting. Previous RWS work found Q5 RSA to be associated with FIM Self-Care and Neuro-QoL Upper-Extremity Function and specifically linked posterior reachability with dressing and toileting performance [19]. In the present study, the association between Q5 RSA and ADEM further suggests that posterior reach capacity may relate to the diversity of spontaneous arm movement in daily life.

Overall, these exploratory findings suggest that regional RSA may capture complementary aspects of upper-extremity function that are obscured by total RSA alone. However, because no pairwise differences in correlation strength were statistically significant after correction, the present data do not establish that any particular clinical or wearable metric is preferentially associated with a specific workspace region.

### 4.5. Limitations and Future Work

This exploratory study was limited by its small sample size, which reduced statistical power, increased uncertainty around the estimated associations, limited adjustment for potential confounding factors, and precluded meaningful subgroup analyses. Although the within-participant RWS comparisons and rank-based association analyses were appropriate for the available cohort, the findings should not be interpreted as definitive population estimates or evidence of causality. Recruitment was also constrained by the burden of the parent 9-week protocol, which required repeated clinical assessments and prolonged home smartwatch monitoring. Larger and more diverse cohorts are needed to estimate these associations more precisely, evaluate their robustness, and determine their generalizability. Because wearable monitoring was limited to a single smartwatch worn on the paretic arm, relative arm-use ratios and interlimb asymmetry could not be quantified. Future studies should incorporate simultaneous bilateral monitoring to help distinguish whether changes in relative use reflect increased paretic-arm activity, decreased contralateral-arm activity, or broader changes in overall activity. Bilateral monitoring could also provide information about compensatory reliance on the nonparetic arm and simultaneous use of both arms during daily life.

Future work should examine whether smartwatch-derived orientation, elevation, and movement trajectories can be used to estimate a wearable-based reachability or spatial-coverage score such as RSA without requiring a depth camera. Such a measure would need to be validated against camera-based RSA but could support repeated and scalable assessment in the home. The feedback system could also be redesigned to target reachable workspace directly by encouraging movement into underused upper, lateral, or posterior regions rather than focusing only on GMAC or ADEM goals. Larger longitudinal studies should test whether spatially targeted feedback, an approach that shows promise for improving planar, supported reaching [60], improves both clinic-based RSA and real-world arm use. Although RWS and smartwatch monitoring characterize related but distinct aspects of upper-extremity function, the present study did not evaluate whether considering both measures improves clinical assessment, decision-making, or patient outcomes beyond either method alone. Future prospective studies should evaluate whether their combined use provides incremental clinical value for personalized rehabilitation.

## 5. Conclusions

The study’s findings support RWS as a valid and relevant measure of arm impairment, with greater reachable workspace associated with less upper-limb impairment, better gross manual dexterity, and greater self-reported amount and quality of paretic-arm use. For the first time, camera-based RSA was also related to wrist-worn measures of real-world arm performance, with ADEM showing significant associations with reachable workspace. This suggests that daily time spent moving in diverse ways may provide useful information about how clinic-based spatial capacity is expressed during daily life. Together, RWS and ADEM may help bridge clinic-based assessment of upper-limb capacity with continuous measurement of real-world performance after stroke.

## Figures and Tables

**Table 3:** Comparisons of dependent correlations between Total Relative Surface Area and paired clinical, self-reported, and smartwatch-derived outcomes. Correlations are reported as Spearman’s ρ. The difference in correlation strength, Δρ, was calculated as the Total RSA correlation with the first outcome minus its correlation with the second outcome. Williams tests compared dependent overlapping rank correlations, and their *p*-values were adjusted across the three comparisons using the Benjamini–Hochberg procedure. Confidence intervals are participant-level 95% BCa bootstrap intervals based on 10,000 paired resamples.

Outcomes	Δρ	95% CI for Δρ	Williams t	df	*p*	BH-Adjusted *p*
BBT versus UEFM	0.001	[−0.32, 0.52]	0.003	11	0.997	0.997
MAL-AOU versus MAL-QOM	0.19	[−0.02, 0.86]	2.126	11	0.057	0.086
ADEM versus GMAC	0.19	[0.01, 0.78]	2.123	11	0.057	0.086

## Data Availability

The datasets generated or analyzed during this study are available from the corresponding author on reasonable request.

## Abbreviations

The following abbreviations are used in this manuscript:

ADEM	Active and Diverse Exploratory Movement
ADL	Activities of Daily Living
AIC	Akaike Information Criterion
ARAT	Action Research Arm Test
BBT	Box and Block Test
FIM	Functional Independence Measure
FSHD	Facioscapulohumeral Muscular Dystrophy
GDS	Geriatric Depression Scale
GMAC	Gross Movement and Activity Count
IMU	Inertial Measurement Unit
IQR	Interquartile Range
MAD	Median Absolute Deviation
MAL	Motor Activity Log
MAL-14	14-item Motor Activity Log
MAL-AOU	Motor Activity Log Amount of Use
MAL-QOM	Motor Activity Log Quality of Movement
Neuro-QoL	Quality of Life in Neurological Disorders
PRO	Patient-Reported Outcome
QoL	Quality of Life
QuickDASH	Quick Disabilities of the Arm, Shoulder and Hand
Q1	Upper medial reachable workspace quadrant
Q2	Lower medial reachable workspace quadrant
Q3	Upper-lateral reachable workspace quadrant
Q4	Lower-lateral reachable workspace quadrant
Q5	Posterior lower-lateral reachable workspace quadrant
ROM	Range of Motion
RSA	Relative Surface Area
RWS	Reachable Workspace
UE	Upper Extremity
UEFM	
WMFT	Wolf Motor Function Test

## Appendix A

The impaired side showed limited evidence of RWS changes when challenged with loading condition. When all participants were included, adding a 500 g distal load (wrist weight) did not significantly alter most quadrant-level RSA scores, although Q4 showed a significant reduction in the weighted condition (*p* = 0.030). After excluding MAD-flagged paired-difference outliers, the Q4 comparison no longer reached significance but showed a similar trend (*p* = 0.054). In contrast, Total RSA became significantly lower in the weighted condition after outlier removal (*p* = 0.007), suggesting that added weight may have a modest effect on overall reachable workspace. However, because no individual quadrant remained significant after outlier removal and because the 500 g load was 10 times heavier than the smartwatch itself, the main analysis from this paper focused on the no-weight RWS condition.
